# Are falls the health concern only for the older population?

**DOI:** 10.1016/j.lanwpc.2021.100186

**Published:** 2021-07-06

**Authors:** Pengpeng Ye

**Affiliations:** a The George Institute for Global Health, University of New South Wales, Sydney, Australia; b National Centre for Non-Communicable Disease Control and Prevention, Chinese Centre for Disease Control and Prevention, Beijing, China

Falls had been recognized as a complex but preventable health issue amongst older people [1]. The Global Burden of Disease (GBD) study 2017 showed an increased incidence, disability-adjusted life years and mortality of falls among older people during the last three decades [2]. Falls also ranked the 16^th^ cause of mortality from 293 diseases and were the leading cause of injury-related mortality among Chinese older people in 2017 [3]. Compared to abundant researches stressing the burden of falls among the older population, limited studies have highlighted the falls, particularly for those warranting medical attention, as an emerging health issue in the younger population [4]. In a recent technical package published by World Health Organization (WHO), children, adolescents and worker in several high-risk occupants are explicitly identified as the key risk groups of falls [5].

In *The Lancet Regional Health – Western Pacific*, Ching-Lung Cheung and colleagues analysed the secular trends in fall-related hospitalizations in adolescents, youth and adults from Hong Kong between 2005 and 2018 [4]. By analysing a total of 336 439 patients aged 10 years and above with fall-related hospitalization during the 14-year study period, the authors showed a significant increase of fall-related hospitalization in both the older and younger population [4].

This retrospective cohort study based on a high-quality population-wide electronic database of Hong Kong highlighted the important role of routinely collected data, which could facilitate clinical practitioners and scientists better assess and monitor the epidemiological characteristics of varied health concerns across different populations within diverse socioeconomic context settings and generate evidence to inform clinical practice and public health decision making. As the first population-based study on the incidence of fall-related hospitalization among people aged 10 years and above in Hong Kong, this study also raised the urgent concern of negative impacts of fall-related injuries on both the older and younger population and the awareness of fall-prevention and management efforts in high-risk groups.

This study provided essential fall-related clinical outcomes in line with the study design and the utility of data source and explicitly highlighted the increasing burden of falls among different age groups. These findings implied the potential benefits of widely strengthening fall-prevention and management efforts in current clinical practice, which could also align well with the strategies in falls prevention and management across the life-course and integrated care for older people advocated by WHO [5,6]. This study also noted that the current clinical fall-prevention resources were rarely allocated to the younger population in Hong Kong. Although falls could be a normal part of developing movement and risk-assessment skills among children, adolescents and young adults, fall events with injurious outcomes should not be inevitable. More engagement and efforts should be attained from the frontline clinical staff to prevent falls and reduce the detrimental consequence of falls among the younger population in the life-course perspective.

Compared to most regions and countries, particularly for those in the Western Pacific region, great fall-prevention and management efforts targeting the older population, coupled with policy and financial support, have been being jointly contributed by different stakeholders in Hong Kong during the past decade [7]. However, the increasing trend of fall-related hospitalizations was still identified in this population-based study. A counterfactual situation could be naturally and reasonably presumed in the absence of these efforts that there could be more older people suffering fall-related injuries. The conflicting results of multifactorial falls prevention interventions across different researches were also discussed in this study. Therefore, the evaluation of current fall-prevention and management efforts should be conducted to provide more accurate and robust evidence of whether and how these efforts would reduce falls and relevant immediate and long-term consequences among the older population in Hong Kong. As for the younger population, more efforts could be aggressively expected in clinical researches including, but not limited to, risk factors and epidemiological pattern analysis of falls and fall-related injuries, the impact and cost-effectiveness evaluation of fall-prevention and management efforts in clinical practice, and barriers and facilitators identification of implementing falls prevention interventions in different clinical settings.

There remain some unanswered questions from a clinical research perspective. First, this study has reflected a relatively comprehensive situation of hospitalized fall patients in Hong Kong, but it did not capture those seeking medical care due to falls in outpatient clinics of public hospitals, patients with falls admitted to private hospitals, and patients suffering falls within the hospital. Second, all-cause mortality adopted in this study could avoid the potential underreporting of falls due to the difference in death cause coding practice, but whether it would overestimate the burden of falls in terms of clinical outcomes and compromise the implications for clinical practice and public health system needs to be explored further. Third, if a detailed cost analysis of fall-related hospitalizations could be provided in the study, the potential audience could have a better understanding of the impact of falls on clinical practice and the public health system. Fourth, more efforts could be expected to harness the power of advanced analysis methods, e.g., data mining and knowledge graph, to bridge the gap of the available data and the best information on falls among the population in Hong Kong.

Generally, this study gave us a clear answer to the question “Are falls the health concern only for the older population?” using a population-based electronic medical database in Hong Kong. Three important take-home messages could be summarized for all concerned audiences and also extended to regions and countries experiencing a similar situation of falls among the population. First, falls are a growing health concern, not only for the older population but also for the younger age groups. Second, despite increased fall-prevention and management efforts made in clinical practice and the public health system, the increasing burden of falls could be still projected in different income and resource settings. Third, sustained, targeted and coordinated falls prevention strategies across the life-course are urged from multisectoral collaborations and multidisciplinary commitments to address falls on a local and global scale.

## Declaration of Competing Interest

Pengpeng Ye is supported by the Dr John Yu Fellowship from the George Institute for Global Health and Tuition Fee Scholarships from University of New South Wales. The author declares no competing interests.
